# Patriarchal Forestalling—the Anticipatory Structure of Legal Failure on Violence against Women

**DOI:** 10.1093/ojls/gqag014

**Published:** 2026-04-01

**Authors:** Shazia Choudhry

**Keywords:** patriarchal forestalling, structural injustice, domestic violence, epistemic injustice, feminist legal theory, India

## Abstract

This article introduces the concept of patriarchal forestalling to explain why formally rights-protective systems frequently deliver recognition without remedy in cases of domestic abuse. Forestalling denotes an anticipatory repertoire through which legal and paralegal institutions diffuse and neutralise claims via five recurrent dynamics: normative socialisation, relational surveillance, pre-emptive discrediting, institutional redirection and procedural dereliction. Drawing on British Academy-funded qualitative research conducted across Maharashtra, Tamil Nadu and West Bengal, the article demonstrates how these mechanisms sustain the appearance of responsiveness whilst withholding meaningful protection. Addressed to a general legal readership, the article makes two principal contributions. Conceptually, it shifts the analysis from doctrinal design to the broader institutional ecology within which rights are operationalised. Methodologically, it provides a vocabulary for identifying when practice reconstitutes rights as matters of discretion. Although grounded in the Indian context, the framework travels beyond it, offering insight into how liberal legal orders may affirm rights at the level of principle while remaining structurally configured to forestall gender-equalising change.

## 1. Introduction

This article interrogates the failures of legal responses to domestic violence through the conceptual framework of patriarchal forestalling, a term used to describe how patriarchal institutions systematically pre-empt and redirect women’s resistance, recoding violence as usual, domestic or unworthy of legal remedy. Drawing on the findings of a British Academy-funded joint qualitative research project conducted across three Indian states,^1^ the study illustrates how patriarchal forestalling operates across five dynamics: through normative socialisation, which frames endurance as virtue; through relational compliance, by which family and community actors’ police the choices, movement and credibility of women; through pre-emptive discrediting, where survivors’ narratives are met with suspicion; through institutional redirection to community-based mechanisms that lack gender-sensitive mandates; and through procedural dereliction, whereby justice is rendered inaccessible via delays, weak enforcement or non-compliance.

These dynamics, I argue, are not merely anecdotal or accidental. They are systemic outcomes of a justice apparatus deeply embedded within patriarchal logics. These logics privilege familial stability over individual rights, male authority over female autonomy and societal order over transformative accountability. This article draws attention to the intersectional exclusions faced by Dalit, LGBTI, migrant and economically marginalised women, whose experiences are further erased or trivialised within dominant institutional responses. In highlighting these structural barriers, the analysis reaffirms that justice for survivors of domestic violence in India remains not only elusive, but actively obstructed, more rhetorical than real. By foregrounding the anticipatory, normative and systemic mechanisms of patriarchal control, I argue for a shift away from technocratic assessments of implementation towards a more critical understanding of the ideological foundations that sustain gendered injustice.

Set against the global landscape, the argument addresses widely recognised gaps between law on the books and law in action. International legal frameworks, most notably the Convention on the Elimination of All Forms of Discrimination against Women (CEDAW) and the due diligence standard articulated by the UN Special Rapporteur on violence against women, make it clear that states must prevent, protect, prosecute and provide reparation, thereby embedding accountability for acts of both commission and omission.^2^ This due diligence obligation requires states to ensure effective prevention, protection, prosecution and reparation, thereby embedding state accountability for acts of commission and omission in cases of gender-based violence.^3^

Many jurisdictions have responded: as of 2021, over half of all economies (104 out of 190) had adopted comprehensive domestic violence legislation.^4^ Yet significant gaps persist in implementation and enforcement,^5^ undermining the United Nations’ Sustainable Development Goals of gender equality, good health and well-being. A 2022 World Bank assessment found that only 38% of the policy frameworks necessary for implementing gender-based violence legislation were in place globally, with significant disparities in service provision, monitoring, coordination and budgets.^6^ These challenges are particularly acute in South Asia, where over 60% of countries lack the institutional frameworks required to enforce gender-based violence laws and where resource allocation is amongst the lowest globally.^7^ The stakes of the implementation gap are underscored against the backdrop of the estimates of prevalence of the World Health Organization (WHO): nearly one in three women worldwide experience physical and/or sexual violence in their lifetimes, with exceptionally high regional rates and intimate partners responsible for a large share of female homicides.^8^

India, despite having made considerable legal progress, most notably through the Protection of Women from Domestic Violence Act (PWDVA) of 2005, remains a paradigmatic example of the disjuncture between legal commitment and substantive justice. The PWDVA was a landmark in recognising not only physical, but also emotional, sexual, verbal and economic, abuse within domestic relationships.^9^ Yet, implementation has been uneven and inconsistent across states, marred by bureaucratic inertia, poor inter-agency coordination and a lack of dedicated funding.^10^

Although grounded in the Indian legal context, the analysis offered here, particularly the conceptualisation of patriarchal forestalling, has broader jurisprudential implications. It intervenes in ongoing debates about the structural limits of legal reform in contexts of deeply embedded gendered inequality. It also raises critical questions about the normative and institutional conditions under which law purports to deliver justice.^11^ The project findings reveal how legal mechanisms that are formally rights protective may nonetheless function in practice as sites of anticipatory containment, redirecting or diffusing women’s claims through a constellation of procedural, epistemic and moral strategies.

This article thus contributes not only to the literature on domestic violence and legal implementation, but to broader legal theoretical concerns about the disjunction between legal form and substantive justice. For a generalist legal readership, the analysis advances a diagnosis of how law, when embedded in patriarchal structures, may enact a simulation of remedy and maintain the performance of responsiveness whilst structurally precluding transformation. In this respect, the Indian case study operates not as an outlier, but as a critical exemplar of how legal institutions can reproduce hierarchies despite attempts at reform. The concept of patriarchal forestalling provides a theoretical vocabulary through which to interrogate the recursive and anticipatory nature of legal failure, inviting reflection on the deeper normative and institutional recalibrations required for justice to become more than a symbolic promise.

The body of the article proceeds in four main sections. Section 2 outlines the methodological approach, detailing the qualitative research conducted across multiple Indian states. Section 3 introduces the legal and policy context in India concerning domestic violence and abuse. Section 4 provides the conceptual and theoretical grounding for the analysis, presenting the framework of patriarchal forestalling to explain how gendered institutional logics pre-empt, defer or disqualify women’s claims to justice. It situates this within broader feminist critiques of state complicity, structural injustice and the normative preservation of patriarchal order. Section 5 presents the core empirical findings. It identifies and elaborates on the five key dimensions of patriarchal forestalling: normative socialisation through familial duty; relational compliance by family and community actors; pre-emptive discrediting of survivors; institutional redirection to informal forums; and procedural dereliction through bureaucratic delay and uneven implementation. Each dimension is illustrated with narrative evidence from survivor accounts. Finally, the article concludes by reflecting on the implications of patriarchal forestalling for legal reform and feminist justice.

## 2. The Research Project: Methodology and Research Design

The data upon which this analysis is based is drawn from a British Academy-funded multidisciplinary research project which sought to examine the persistent barriers to realising the legal protections and support mechanisms for survivors of domestic violence in India. It also sought to interrogate the disjuncture between formal legal commitment and the realities of substantive justice through an academic–civil society research collaboration. Drawing on qualitative fieldwork conducted across three Indian states—Maharashtra, Tamil Nadu and West Bengal—between July 2021 and April 2022, the study spans rural, semi-urban and urban sites in each state. It explores how survivors and those around them understand domestic violence and legal rights, their experiences of seeking help and justice, and the formal and informal pathways through which they attempt to mitigate, endure or exit violent relationships. Central to the inquiry is an examination of what unfolds in the space between the experiences of harm and the law’s uneven, biased or indifferent response, and whether these legal journeys yield any meaningful benefits for those who undertake them.

The three Indian states of Maharashtra, Tamil Nadu and West Bengal were chosen largely because the study was built around established, state-based research teams and civil-society partnerships that could recruit participants, conduct interviews and handle translation/transcription locally. Methodologically, using three states also supported comparison across distinct regional contexts whilst keeping the design consistent within each state (rural, semi-urban and urban sites), strengthening the project’s ability to examine how survivors navigate different local service and governance ecosystems.

The study drew on qualitative research across three participant groups. Semi-structured interviews were conducted, one on one, with 180 survivors of domestic violence (60 in each state), across rural, semi-urban and urban sites. All interviews were conducted in local languages (Marathi in Maharashtra, Tamil in Tamil Nadu, Bengali in West Bengal).

Using an intersectional sampling approach, the research team purposively selected women from diverse caste, class, religious, sexual and disability backgrounds, although questions on caste, sexual orientation and disability were not universally answered, possibly because of the sensitive nature of these categories within the Indian context.^12^ Participants were recruited through mapping exercises, existing support networks and snowball sampling. The interviews explored experiences of violence, help-seeking strategies and legal awareness. A further 180 interviews were conducted with local stakeholders, including lawyers, healthcare professionals, non-governmental organisation (NGO) workers, police and protection officers, who play key roles in implementing the PWDVA and supporting survivors. These interviews examined how domestic violence is understood, how the law is interpreted in practice and what obstacles exist in service delivery and legal enforcement.

Lastly, 90 interviews were conducted with community members in each region. Participants included individuals from a wide range of occupations, such as auto rickshaw drivers, homemakers, shopkeepers, teachers and craftspeople, offering insight into local norms and attitudes towards domestic violence. These interviews did not explore individuals’ own experiences of abuse but instead focused on public understandings and informal support systems. The resultant data was analysed using a coding protocol and with the assistance of ATLAS.ti software.

The research adhered to WHO ethical protocols for the study of domestic violence.^13^ A key limitation lies in the sampling of survivors primarily through support organisations, meaning that participants were more likely to have recognised their experiences as abuse and to have accessed help. As such, the findings likely reflect a greater degree of legal awareness and engagement than is present in the broader population captured by national survey data.

## 3. Law, Violence and the Limits of Legal Reform in India

The prevalence of domestic violence in India is high. Crimes against women rose by 26.35% over six years to 428,278 cases (64.5 per 100,000), with domestic violence comprising the largest share. According to the National Family Health Survey of 2020–21 (NFHS-5), 29.3% of ever-married women aged 18–49 reported domestic and/or sexual violence, and 3.1% of pregnant women in this age group reported physical violence during pregnancy. Intimate partner violence is pervasive: 31.9% of ever-married women aged 18–49 reported emotional, physical or sexual violence by husbands, with a prevalence of 27.3% in urban areas and 34.0% in rural areas.^14^ In the Crime in India 2022 statistics (police-recorded),^15^ there were 448,211 total crimes against women (rate 66.2 per lakh female population), the largest single category of which was ‘cruelty by husband or his relatives’ (Indian Penal Code (IPC) (1983), section 498A), with 133,676 cases (about 29.8% of crimes against women; rate 19.7 per lakh). In parallel, the National Commission for Women^16^ complaint data, reflecting help-seeking through the Commission rather than FIR registration, recorded 28,811 complaints of crimes against women in 2022, including 6274 complaints categorised as domestic violence. Together, these figures highlight both the scale of police-recorded intimate-partner/family cruelty and the continued volume of non-police complaint pathways. It should also be noted that these are reporting/administrative counts rather than prevalence estimates.

Over recent decades, India’s response to domestic violence has evolved in response to grassroots feminist pressure and international norms.^17^ Section 498A of the IPC^18^ criminalised ‘cruelty’ within marriage, but significant gaps remained: sexual and economic abuses were not recognised; coverage was limited to legally wedded spouses; and there were no civil protections for residence, maintenance or exit, leaving many women to choose between a criminal complaint and economic security.^19^ The PWDVA addresses these limits by reframing domestic violence as a public concern and introducing a civil protective scheme alongside criminal law.

The legislation also introduced several key legal reforms. The PWDVA widens ‘domestic relationship’ to cover anyone who lives or has lived in a shared household, whether related by consanguinity, marriage, a relationship ‘like marriage’, adoption or as members of a joint family.^20^ It broadens the definition of ‘domestic violence’ to include physical, sexual, verbal/emotional and economic abuse (eg deprivation, disposal, or restriction of access to resources) and establishes a civil protective scheme, protection, residence, and monetary relief/maintenance orders (with custody/compensation where appropriate) that operates alongside criminal law (eg IPC, section 498A),^21^ and makes the breach of a protection order a criminal offence. Of particular significance is the creation of a coordinated response architecture, recognising service providers and self-help groups, and designating protection officers (POs) as the first point of contact to link survivors to medical, residential and legal support. Finally, it mandates periodic sensitisation and training for central and state officials, including police, healthcare providers and the judiciary.^22^

However, despite statutory reform, implementation has faltered due to several structural failures, the first being the inability to ring-fence central funding.^23^ In the absence of national provision, states have proceeded unevenly, with some yet to operationalise the scheme. A recent Oxfam budget-tracking study^24^ reported that only One-Stop Centres and Swadhar Greh directly support the PWDVA’s mandates, and even these are underfunded, resulting in an estimated 85% annual shortfall for women-specific response services. Second, chronic underinvestment in legal aid and court infrastructure has made the statute’s fast-track aspirations unattainable. Third, the linchpin role of POs remains unrealised: officers are formally appointed in only seven states. As of 2013, from the last available national monitoring available, there were 6279 POs nationwide (for a 2011 population of 586.46 million), and only 27.6% were women, despite the PWDVA’s preference.^25^ In addition, mandatory sensitisation has been sporadic, leaving gender-conservative norms intact; in practice, POs are often drawn into untrained ‘counselling’ that moralises endurance and family preservation.^26^ The legislation also omits sexual minorities and provides inadequate accommodation for disabled survivors.

Lastly, comprehensive monitoring of the PWDVA is largely absent, as there has been no national reporting architecture and no countrywide tracking since the Lawyers Collective’s fifth Staying Alive report in 2013. The limited official figures available on the PWDVA, which capture only criminal cases relating to breaches of protection orders, continue to point to extremely weak criminal enforcement, substantial attrition and backlog. Earlier NCRB data recorded 553 cases in 2019, 446 in 2020 and 507 in 2021. The most recent Crime in India bulletin, published in 2023,^27^ shows that enforcement remains minimal: during 2023, police investigated 656 PWDVA cases, only 299 of which were disposed of at the police stage. At the court level, only 17 convictions were recorded nationwide in 2023 (including cases from previous years), alongside seven discharges and 80 acquittals. By the end of the year, 3486 cases remained pending trial, translating into a pendency rate of 96.8%, with convictions occurring in just 16.3% of completed trials.

The lack of up-to-date data and concerns around the lack of sufficient POs, service providers and shelter homes, in addition to long delays, have recently been criticised by the Supreme Court of India. As a result of a petition filed by an NGO seeking implementation of the PWDVA,^28^ the National Legal Services Authority was instructed to provide up-to-date data. As of July 2022, more than 471,000 PWDVA matters were pending, with 21,008 appeals outstanding.^29^ Together, the absence of a reporting regime and the scale of the backlog underscore a systemic capacity shortfall that renders the PWDVA’s guarantees unrealisable.^30^

Structural issues aside, societal attitudes also present a significant challenge. NFHS-5 data indicate entrenched normative acceptance of abuse: nearly half of respondents (45.4% of women and 44.3% of men) consider a husband justified in beating his wife in at least one of seven scenarios.^31^ Predictably, help-seeking is rare: 77% of women neither disclosed nor sought help (only 14% said they did), with non-disclosure higher in rural than urban areas (78.6% v 73.2%). When assistance is sought, it is overwhelmingly informal, with family (58.3%), husband’s family (28.1%) and friends (18.7%) being the most common sources. Formal avenues central to the PWDVA remain marginal, with representation from police (8.2%), social services (2.2%), religious leaders (2.0%), health professionals (1.7%) and lawyers (1.6%). In addition, urban women are somewhat more likely than rural women to seek help from any source (17.2% v 13%).^32^ These patterns illustrate how, despite the PWDVA’s presence, the social legitimisation of violence suppresses recourse to institutions and simultaneously undermines progress towards SDG targets on gender equality and health.^33^

Due to space limitations, it is not possible to provide a detailed account of state-level policy responses or disaggregated domestic violence data. Suffice to say, the comparative context across the three states studied in the research project reveals several significant patterns. NFHS-5 data^34^ indicate that most women in these regions neither disclose experiences of domestic violence nor seek formal support; when they do seek help, they are far more likely to turn to family or friends than to the police, legal professionals or medical providers. Tamil Nadu demonstrates extremely low engagement with formal justice mechanisms: only 2.3% of women report violence to the police, and less than 1% consult lawyers. Social attitudes further compound these institutional barriers: across all three states, significant proportions of women express acceptance of wife beating under certain conditions, with Tamil Nadu exhibiting the highest levels of justification. Whilst Maharashtra demonstrates relatively greater investment in service provision compared to West Bengal and Tamil Nadu, the NCRB data nonetheless point to a shared pattern across all three states of limited criminal enforcement capacity, low prosecution throughput and persistent judicial backlog in domestic-violence-related cases.

This pattern is reflected in police-recorded data from the Crime in India 2023 tables.^35^ Maharashtra and West Bengal continue to rank among the highest-reporting states for crimes against women, with Maharashtra recording over 56,000 cases and West Bengal over 42,000 cases in 2023, while Tamil Nadu reports a substantially lower volume (under 7000 cases), consistent with NFHS-5 findings that only 2.3% of women report violence to the police and under 1% consult lawyers. At the level of criminal enforcement under the PWDVA, statewise court-disposal tables show extremely small numbers of trials concluded and convictions recorded in each of the three states, alongside very high pendency, indicating that breaches of PWDVA protection orders rarely translate into completed criminal proceedings.

## 4. Patriarchal Forestalling: Concept and Structure

Building on the implementation record outlined in section 3, where the PWDVA’s formal guarantees are not being realised due to uneven roll-out and weak enforcement, this section provides an explanatory lens, patriarchal forestalling, to explain why these failures occur. A good deal of research has demonstrated how victims of domestic abuse are subject to systemic injustice; the deeply embedded and institutionalised forms of injustice that arise from the structures, policies, norms and power relations that govern society.^36^ In the context of victims of domestic abuse, systemic injustice operates not through isolated incidents, but through the routine functioning of social, legal and political systems that fail to protect or actively harm survivors. These unjust systems are both pervasive and enduring; understanding the mechanisms that underpin their persistence is therefore crucial if they are to be dismantled.

Haslanger^37^ provides a compelling explanation of these mechanisms. They argue that these systems are composed of interlocking social spaces, ie multiple institutions and norms that coordinate to uphold injustice and practices that systematically generate and reinforce inequality, harm and deeply embedded patterns of oppression and domination. Injustice is therefore structured into how society functions; it is not an accident, but an outcome of how roles, expectations and institutions are organised. Crucially, such systems are self-perpetuating;^38^ they impose constraints on individual and collective agency in ways that compel the reproduction of the very structures that sustain them. This dynamic, which Haslanger terms a ‘causal loop’,^39^ lies at the core of the difficulty inherent in dismantling or transforming entrenched social hierarchies. From birth, individuals are socialised, through behavioural norms, institutional design, public policy and legal frameworks, into reproducing unjust systems.

Extending Haslanger’s notion of self-reproducing systems, Dembroff contends that unjust social systems are constituted by causal structures oriented towards the reproduction of distinct ideological formations, such as those underpinning gender and racial hierarchies. These ideologies are embedded within and reproduced through the everyday organisation of social spaces and practices. Identifying an unjust system is therefore critical because they do vital explanatory work: it helps us predict, identify and explain various patterns of wrongful treatment, patterns that reflect (actual or perceived) features. This enables us to more accurately determine the types of actions or policies that will most effectively alter those patterns in the future.^40^ ‘Patriarchy’, for example, names the system responsible for systemic gender injustice, ‘white supremacy’ names the system responsible for systemic racial injustice, and so on. Crucially, these structures can function plurally, simultaneously reproducing multiple ideological regimes (eg patriarchy and white supremacy), thereby necessitating the recognition of multiple, analytically distinct systems of injustice. The concept of ‘white supremacist patriarchy’ thus exemplifies how such systems can intersect and co-constitute one another, reinforcing complex matrices of domination. The recognition and analysis of ideologically distinct yet intersecting systems of injustice thus allows for a more nuanced and theoretically robust apparatus for diagnosing and contesting systemic forms of harm.

Patriarchy is understood here as a structural condition embedded in social, legal and institutional arrangements that both enable and obscure male dominance. This builds on the work of earlier feminist theorists^41^ who not only framed patriarchy as a system of interlocking social structures, but also underscored its ideological and cultural dimensions. Dembroff’s articulation of patriarchy,^42^ however, is as a causal structure sustained by normative loops, thereby explaining its persistence not through individual intent, but through socially conditioned role performance and institutional inertia. It also emphasises that patriarchy operates not only through overt violence or explicit discrimination, but through everyday practices, beliefs and institutional arrangements that shape and constrain the lives of those gendered as women and marginalised. These insights compel a broader jurisprudential reflection: if legal systems are among the institutions that constitute these ‘causal loops’, then the very design and administration of justice must be interrogated as structurally implicated in the reproduction of inequality. Dembroff thus defends the coherence of patriarchy as an autonomous system of injustice,^43^ offering a unique explanatory framework for understanding the regulation of individuals through gendered norms and ideals. This conceptual differentiation is not merely theoretical; it has practical implications for social movements, as it enables the identification of specific mechanisms of oppression and thereby facilitates targeted strategies for resistance and transformation. The failure to individuate unjust systems, Dembroff argues, thus risks obfuscating the modalities of domination that liberation movements seek to contest, thereby hindering their ability to focus their resources and efforts effectively.

Developed in response to the key themes that emerged from the empirical evidence gathered across three Indian states, this article builds on Dembroff’s work and feminist and legal critiques^44^ concerning epistemic injustice by formulating the concept of *patriarchal forestalling* to demonstrate how legal institutions may be shaped not only by inertia or neglect, but also by anticipatory rationalities, internalised practices and procedural defaults that work to disarm resistance before it becomes juridically legible. In this sense, the concept offers more than a sociological account of gendered violence: it names a failure of legal recognition, legal responsiveness and, ultimately, legal imagination.^45^ This framing not only reveals the hidden scaffolding of power behind domestic violence, but also clarifies how legal reform without systemic change remains insufficient.

Patriarchal forestalling also names and theorises the patterned ways in which patriarchal structures pre-empt, obstruct or deflect survivors’ pathways towards justice and redress. Where Dembroff argues that patriarchy constitutes a structurally unjust system, maintained through formal and informal institutions that naturalise gender hierarchies, the framework captures a more dynamic, anticipatory dimension of this system. It elucidates how patriarchal institutions actively neutralise potential disruptions and foreclose transformative outcomes through the social, institutional and procedural reabsorption of survivor agency, through which women’s efforts to resist violence or claim justice are not openly rejected but instead absorbed back into normative systems that maintain patriarchal control. Socially, this occurs through moralising discourses of adjustment, sacrifice and family honour that pressure survivors to reinterpret resistance as deviance and endurance as virtue. Procedurally, the legal system enacts a subtler form of containment, through delays, evidentiary burdens and non-enforcement, that deters women from pursuing their claims. This dynamic reflects a broader logic of anticipatory governance, whereby legal institutions are structured not merely to adjudicate claims after harm has occurred, but to manage and pre-empt perceived disruptions to the normative social order, particularly those posed by survivor resistance to patriarchal authority. In each case, survivor agency is not extinguished but strategically redirected into channels that reaffirm the very hierarchies’ survivors seek to escape. This reabsorption exemplifies the anticipatory logic of patriarchal forestalling: systems are pre-emptively structured to manage and neutralise dissent, ensuring that efforts to transform injustice are repackaged as conformity, endurance or private reconciliation. In this sense, it is a system of recursive containment, where each step survivors take towards liberation is absorbed and redirected in ways that preserve existing gender hierarchies.

## 5. From Framework to Findings: The Five Dynamics in Practice

To frame the analysis that follows, I employ patriarchal forestalling to describe an anticipatory repertoire through which legal and paralegal institutions neutralise challenges to the gendered order before they mature into enforceable claims. The repertoire comprises five recurring dynamics. Normative socialisation encodes endurance as virtue and domestic harm as ordinary, shaping what can be named as violence and when. Relational compliance mobilises family, neighbours and community authorities to police conformity through a moral vocabulary of honour, duty and adjustment. Pre-emptive discrediting installs a credibility deficit at the point of uptake, recasting women’s testimony as suspect and burdening it with unattainable evidentiary demands. Institutional redirection translates complaints into matters for conciliation, diverting them into forums oriented to reconciliation rather than remedy. Procedural dereliction then converts rights into delay, through missed timelines, adjournments and weak enforcement, preserving recognition whilst withholding effect. These dynamics are iterative and mutually reinforcing: the first two shape the conditions of disclosure; the middle two gatekeep entry and trajectory; and the final one drains momentum from within law. They should be read not as a rigid sequence, but as a modular architecture that sustains the appearance of responsiveness while safeguarding the status quo. The following subsections take each dynamic in turn, clarifying its distinctive operation and its role in producing symbolic recognition without protection.

### A. Normative Socialisation

Normative socialisation names the processes through which women and men learn the moral logics that render intimate partner violence thinkable, tolerable and, up to a point, expected. These are not simply private beliefs; they are socially taught scripts of gender propriety that are affirmed in everyday life and retrospectively naturalised as common sense. In Kandiyoti’s terms, these scripts organise the ‘bargains’ through which women secure recognition and survival within patriarchal orders; the bargain is moral before it is strategic, and it sets the parameters of what counts as a *reasonable* response to harm.^46^ Survivors are thus persuaded, often by family, professionals and broader social discourse, that their suffering is a moral obligation tied to their gender. As a result, violence is not named as injustice but is instead reframed as a test of feminine virtue, marital endurance or family honour. The imperative to ‘adjust’, ‘endure’ or ‘keep the family together’ operates as a moral script that renders resistance deviant, selfish or dishonourable. This normative logic works by internalising patriarchal values, so that complicity becomes not just socially expected, but ethically valorised.

Within this dynamic, women learn, long before any encounter with police, lawyers or courts, what kinds of suffering are expected, what forms of endurance are virtuous and when, if ever, complaint is morally justified. The effect is to normalise violence as ‘discipline’ or a private ‘family matter’, recoding it as integral to household order rather than as a wrong that calls for redress. Connell’s account of hegemonic masculinity further clarifies this mechanism by demonstrating how culturally exalted forms of manhood (authority, control, discipline) are embedded in household and kinship organisation, making violent ‘correction’ appear protective rather than coercive.^47^

Within this frame, formal instruments such as the PWDVA enter a terrain in which family sanctity is treated as paramount and the idiom of adjustment, accommodation and silence is already dominant. This is exacerbated by the construction of gender in India, in which, through a complex interaction of family, religion, law and societal norms, women’s roles are predominantly defined by marriage and motherhood; their identities deeply entwined with their relational duties to men. In contrast, men’s roles are defined by dominance, protection and control.^48^ As a result, domestic violence forms a constitutive element of everyday domestic life, functioning as a tacitly accepted practice that maintains and reinforces patriarchal structures in a broader landscape of state and societal gendered violence.^49^ Legal frameworks, such as the PWDVA, have sought to intervene in this space, but enforcement remains inconsistent, hindered by the state’s reluctance to disrupt familial sanctity.^50^

The project findings consistently demonstrate these dynamics. Across states and sites, survivors reported intersecting physical, psychological, economic and sexual violence. Perpetrators were principally husbands, but also marital family members, especially mothers-in-law, and, less often, natal family. These patterns, as indicated by previous research,^51^ cut across caste and class, urban and rural contexts, undermining the stereotype that domestic violence is confined to poorer or rural households. The common thread was not demographic location but a normative order that renders intimate violence an ordinary, indeed constitutive, feature of domestic life,^52^ helping to explain persistently low levels of help-seeking despite legal reform.

Survivors repeatedly reported that a baseline of violence was coded as ordinary and even morally appropriate within marriage. Some women did not initially recognise partners’ or families’ behaviour as violence or as a violation of rights, describing instead an expectation that the situation would ‘settle down’ or, as Vinita (McS05, rural Maharashtra) recalled being told, ‘women have to bear all this’. Fauzia (McS02, rural Maharashtra) linked her initial reluctance to complain to conditioning: ‘the way we are conditioned, it was hard to complain about any suffering’. Such statements are not idiosyncratic; they index the moral pedagogy through which endurance becomes a measure of feminine virtue. Whilst natal families were frequently the first point of recourse, their support was ambivalent. Women were often encouraged to return to abusive environments to preserve marital stability and family honour.^53^ There were many such examples of relational compliance in the data, where survivors were constantly policed by familial expectations and cultural scripts that deny them the moral permission to leave.

Normative socialisation is also internalised as self-blame and moral responsibility. Mita (MaS03, urban Maharashtra) described assuming the burden of endurance in explicitly ethical terms: ‘Mine was the first wedding in the family and the first divorce, so I had taken it upon myself that it was my moral responsibility to manage … I felt it was my mistake … I should make changes in myself.’ Rabia (TbS10, semi-urban Tamil Nadu) reported a similar translation of structural harm into personal duty: ‘My family blames me for marrying him against their warnings and so they say that I must handle it [domestic violence] myself.’ Such expectations are taught across generations. Preeti (MaS05, urban Maharashtra) recalled her mother being told by her father-in-law, ‘So what? He is a man; he needs to enjoy!’—a formula that excuses male absence and sexual privilege while reinscribing female forbearance. Asha (MAS01, urban Maharashtra) captured the injunction’s peremptory form: ‘I am taught this way, I am conditioned this way to adjust with my husband, come what may. My uncle has told me many times, “You will leave his home only when you die”.’ Swidler’s account of culture as a toolkit is apposite: in ‘settled’ times, people reach for familiar strategies of act, adjust, endure and preserve, and those strategies are then read back as evidence of character.^54^

At the same time, the interviews illustrate that normative socialisation can be challenged. Several survivors described moments of rupture, when the moral sense of ‘what a good wife does’ was interrupted by a counterclaim of self-preservation. Across interviews, talk of thresholds structured help-seeking trajectories. Domestic violence was treated as an assumed norm among survivors, community members and stakeholders; only when it became ‘too much’ did intervention appear permissible. Many survivors sought intervention only when violence escalated to an intolerable level, extreme physical injury, discovery of extramarital affairs or perceived threats to children’s well-being. Meenal (McS16, rural Maharashtra) ‘took steps to end it [the marriage], otherwise he would have killed me’. Amina (TaS05, urban Tamil Nadu) recounted being left for dead by her husband. For Shashi (McS07, semi-urban Maharashtra), a night spent hiding in a store cupboard after a severe beating made the danger incontestable. These accounts do not signal a failure of socialisation, but its relative force: only at the edge of crisis does the injunction to endure finally give way.

Normative socialisation also extended into professional spaces that might otherwise be expected to validate harm. In semi-urban Maharashtra, Anaya’s psychiatrist (MbS09) referred to her ‘high-pitched talking’ and how this might upset her mother-in-law. She then went on to reframe conflict with her mother-in-law as a problem of emotional management, advising her to ‘try to adjust’ and to ‘not fight in front of your son’, lest he learn the behaviour. Anaya concluded: ‘Now I have learnt to control myself, to tolerate her.’ The discourse of mental health is thereby recruited into the wider script of adjustment: resistance is pathologised; compliance is the prescription.

Community surveillance also shaped women’s responses to violence; community networks operated as mechanisms of containment, policing both the articulation of harm and the legitimacy of seeking help. Some neighbours, according to Naina (WcS38, survivor, rural West Bengal), ignored abuse as futile to intervene in; others blamed survivors for their circumstances, as in Kakoli’s case (WaS07, semi-urban West Bengal). Lakshmi (TaS14, survivor, urban Tamil Nadu) described how repeated advice to ‘adjust’ led her to disengage from friends, shrinking her support network and closing off avenues of assistance. In these accounts, survivor silence is not evidence of apathy or ambivalence but the cumulative effect of a normatively saturated environment in which speaking risks shame, gossip and withdrawal of support.

The findings also demonstrated the normalisation of harm in LGBTI relationships, illustrating how gendered scripts intersect with sexuality to foreclose recognition. Megha (WaS21, survivor, urban West Bengal) was ostracised by peers who justified the abuse she experienced: ‘Some even justified the violence, saying this was bound to happen because I was a lesbian.’ Although such episodes also exemplify pre-emptive discrediting, they belong here insofar as the legitimacy conditions for complaint are set by prior socialisation: violence directed at a lesbian woman is coded as predictable, not wrongful.

Normative expectations also interact with structural conditions, tightening the grip of endurance. Economic dependence, insecure property rights and the absence of natal support curtail exit options, particularly for Dalit, tribal and minority women.^55^ For women without natal support, such as Mary (TaS04, urban Tamil Nadu), the ‘decision’ to tolerate violence was often a necessity rather than a choice. As Leela (MbS07, semi-urban Maharashtra) put it, ‘I didn’t earn any money, and so I could not live separately’. For Nepali migrants in Darjeeling, such as Kalpana (WcS56) and Savitri (WcS39), the normative injunction to endure is compounded by legal exclusion from state services due to non-citizen status.

Normative socialisation also anticipates and organises the uptake of formal legality. As Merry^56^ demonstrates, rights are vernacularised through local moralities; translation is non-linear and, where marital endurance is valorised, ‘rights talk’ is recast as advice to reconcile. This helps to explain why, even when women were aware of legal options (including the PWDVA), they often describe prolonged, normalised coping before any formal step. The point is not that law is irrelevant, but that learned scripts of propriety prime the encounter with law: what counts as an *appropriate* complaint, which harms deserve attention and when it is respectable to seek it.

Taken together, the analysis suggests that normative socialisation is the first dynamic of patriarchal forestalling: it sets the horizon of permissible action; it makes endurance legible as virtue and complaint as excess; and it prepares the ground on which the further dynamics: relational compliance, pre-emptive discrediting, institutional redirection and procedural dereliction, can operate. In short, normative socialisation is not a backdrop; it is the anticipatory logic that sets the terms on which violence is rendered ordinary and on which subsequent encounters, with family, community and state, will be read and contained.

### B. Relational Compliance

Relational compliance captures the routine, fine-grained ways in which families and communities regulate women’s behaviour through a moral vocabulary of honour, duty and adjustment. Survivors are not only watched, but also judged and corrected for behaviours that deviate from normative femininity, whether that be leaving a marriage, speaking out about violence or seeking institutional support. Rather than operating as raw coercion, this mode of regulation turns morality into method: it frames endurance as virtue, defines exit as dishonour and makes continued support conditional upon visible conformity to marital and familial ideals. In this sense, relational compliance is a constitutive mechanism of the framework: it translates the social scripts internalised through normative socialisation into day-to-day judgments and sanctions that narrow the range of permissible action before law is ever engaged. This form of surveillance operates through emotional blackmail, gossip, ostracism and withdrawal of social support. Importantly, it often comes cloaked in concern or care, rendering the mechanisms of control as intimate and benign rather than punitive.

The project findings also demonstrate how duty is heavily implicated within the terms of ‘help’. Natal or marital family often presented their involvement as assistance while simultaneously requiring the survivor to adjust and restore domestic order. Support, shelter, mediation and even sympathetic listening were tacitly conditional on recommitment to the marriage. This conditionality is not merely pragmatic; it is explicitly moral. It encodes the norm that a ‘good’ wife or daughter-in-law prioritises familial stability over personal safety, a norm that legal-anthropological studies show shapes the everyday uptake of rights.^57^

Community authorities rendered these norms explicit in public moral terms, mobilising the idiom of honour to regulate women’s movement, work and marital decisions; research on caste and community councils has demonstrated how claims to honour (*izzat*) are deployed to police women’s sexuality, mobility and marital choices, and to justify ‘settlement’ over accountability.^58^ Kala (McS1, survivor, rural Maharashtra) recounted:

Even in Jat Panchayat, Panch (members of council) told me to be at home, women could work outside before marriage, but after marriage they should remain at home. I asked them to show me one woman who can do all the things just being at home. I told them to counsel my husband also. However, that was of no use.

The content of the directive (‘remain at home’) matters, as does its public form: a declaration of what honourable femininity requires, anchored in collective authority.

The same vocabulary governed outcomes. Jacob (TaC02, urban Tamil Nadu), a community member serving on a panchayat,^59^ stated that he is against divorce and would ‘always counsel the couple and ask them to go back to living together’. In Kalyani’s case (WbS13, semi-urban West Bengal), the panchayat urged her to return after an incident of violence because it was ‘the first time’. When she considered further action, she feared being labelled a ‘troublesome character’, accused of disrespecting the council and of failing in her duty to preserve the marriage. Here, ‘adjustment’ operates as a test of character: the honourable woman reconciles; the dishonourable one persists.^60^

Relational compliance also works by calibrated withdrawal. Ethnographies of the ‘ordinary’ in South Asia remind us that what appears as a private choice is often the end product of public vocabularies that organise suffering into the acceptable and the unsayable.^61^ Survivors described an arc in which initial sympathy from clubs, *samaj* bodies or extended family shifted to fatigue, criticism or silence when separation or accountability was pursued. Practical assistance, such as childcare, introductions to problem solvers and shelter, became contingent upon visible compliance with the adjustment. Moreover, in contexts of economic dependence or thin social networks, the risk of losing access to these forms of support made staying appear not only cheaper, but also more moral. This is a familiar pattern in the sociology of honour and household economies: the very resources women need to exit are embedded in the relationships that judge exit as betrayal.^62^

Crucially, these moral judgments produce narratives that travel beyond the home or village. Descriptions of a woman as ‘difficult’, ‘disrespectful’ or ‘unwilling to adjust’ precede her into institutional sites such as police stations, legal-aid offices and courtrooms, where they become background common sense. In this way, relational compliance provides the evidentiary atmosphere for the next dynamic: pre-emptive discrediting, where social suspicion is converted into an interpretive frame that makes disbelief appear reasonable and diversion to ‘counselling’ seem prudent.

Lastly, these processes have stratified effects. Where honour and duty are invoked against women who possess few material alternatives, such as those without natal support, those who are economically dependent on marital families, Dalit and migrant women, the sanctions bite harder. Women without natal family support face strong normative pressures to maintain marital ties and have limited access to alternative housing, childcare or mediation, which significantly constrains their ability to resist abuse. Economic dependence on marital families further heightens vulnerability, as reporting violence can result in loss of shelter, livelihood and access to children.^63^ These risks are intensified for Dalit women, who experience domestic violence at the intersection of gender and caste hierarchies, and are more likely to encounter institutional bias or indifference from police, courts and community authorities.^64^ Migrant women, particularly those who migrate for marriage or work, face additional barriers arising from social isolation, weak local networks, language constraints and insecure documentation, all of which limit their capacity to navigate legal and welfare systems.^65^ As a result, the costs of seeking formal remedies are unevenly distributed, with the harshest consequences borne by women who are already socially and economically marginalised. In such settings, the moral vocabulary is not merely rhetoric; endurance is both the respectable course and, often, the only viable one.

### C. Pre-emptive Discrediting

Pre-emptive discrediting names the anticipatory production of disbelief that meets survivors’ claims at the point of institutional intake. Distinct from normative socialisation (which calibrates the moral permissibility of complaint) and relational compliance (which enforces compliance through reputational sanction), pre-emptive discrediting operates at the level of *epistemic reception*: it frames women’s accounts as instrumentally motivated, exaggerated or false before the facts are examined, and thereby narrows both the remedies considered and the procedures activated. In epistemic terms, it installs a credibility deficit as the baseline against which all testimony is filtered.^66^ Moreover, this anticipatory discrediting often draws on gendered, casteist^67^ or heteronormative assumptions that frame survivors as irrational, vindictive or emotionally unstable. It operates in police stations, courtrooms, health facilities and domestic spaces, where survivors are assumed to be exaggerating or misusing legal protections. By pre-emptively undermining credibility, this dynamic forecloses the possibility of survivors being recognised as legitimate epistemic agents.

A recurrent grammar of ‘misuse’ and ‘false cases’ illustrates this anticipatory frame. Police officers at multiple sites represented the PWDVA and related criminal provisions, especially section 498A of the IPC, as weapons of leverage rather than instruments of protection. Tejal (WaPL11, female police officer, urban West Bengal) asked rhetorically: ‘Asking a husband to leave his parents with a threat to file a case under section 498A, wouldn’t you call it a misuse of law?’ Ishwari (TbP01, a female police officer in semi-urban Tamil Nadu) described it as a professional duty to ‘weed out the false cases’. Such characterisations do not neutrally report evidentiary weakness; they *pre-sift* intake by casting suspicion as prudence and disbelief as responsible gatekeeping.

The discursive availability of ‘misuse’ is reinforced by higher court jurisprudence^68^ that has periodically expressed concern about over-criminalisation under section 498A. There have also been instances where judges have made general statements about the alleged misuse of section 498A, thereby discrediting the testimony of the survivors.^69^ Most recently, in *Janshruti (People’s Voice) v Union of India* (2025),^70^ section 498A was constitutionally challenged on the ground that it was being widely misused and should be rendered gender-neutral or struck down. The Supreme Court dismissed the challenge, holding that the mere possibility of misuse does not render a provision unconstitutional and emphasising that section 498A continues to address a deeply entrenched social harm. However, even as the Supreme Court rejected misuse as a basis for invalidation, the persistence of this line of argument in constitutional litigation contributes to an institutional climate in which concerns about misuse continue to serve as a background justification for reluctance to register complaints and enforce them.

Legal professionals echoed the same anticipatory suspicion. Chandrika (TbL04, female lawyer, semi-urban Tamil Nadu) asserted that ‘women misuse the act very frequently nowadays’, estimating that ‘4 out of 5 domestic violence cases are misuse of the law’ and alleging strategic use of the PWDVA ‘to claim maintenance’, even to ‘trap widowers’. Recasting rights claiming as opportunism in this manner thus licenses the withholding of belief, time and, crucially, advocacy. Here, the credibility judgment arrives in advance of case-specific appraisal; it is a category judgment masquerading as a legal assessment.^71^

Minimisation of visible injury provides a second route for pre-emptive discounting. Sona (MaS17, survivor, urban Maharashtra) recounted approaching a female police officer and ‘show[ing] her marks of beating’, only to be told ‘to ignore it since I had to live with the husband’, with the officer adding that ‘her husband too beat her up’. The evidentiary meaning of injury is neutralised, marks no longer corroborate, whilst the normative meaning is inverted: violence is ordinary; complaint is deviant. In this conversion, disbelief does not simply refuse testimony; it redefines what testimony can *show*. Research on trials in India has described analogous moves in which injuries are redescribed as ‘trifling’, or as consistent with conjugal correction, thereby insulating the institution from the obligation to act.^72^

For survivors situated at the intersection of sexuality, migration status and caste, pre-emptive discrediting is overdetermined by identity-based moralisation. Rubina (WaS21, urban West Bengal) reported that her mother ‘physically harmed my partner’ and then went to the police to complain that her daughter’s partner ‘is a lesbian and spoiling my daughter. My daughter was all right before.’ Here, the object of regulation becomes the relationship itself; the survivor’s claim is reframed as familial deviance requiring police validation. That such complaints coexist with constitutional jurisprudence recognising sexual minorities’ dignity underscores the gap between formal rights and credibility economies within frontline institutions.^73^ The point is not that law is irrelevant; it is that disbelief can domesticate legality by recasting protection as policing identity.

A further modality is anticipatory corroboration: where authorities pre-emptively demand evidentiary support that domestic violence survivors are structurally unlikely to possess. Tapashi (WaPL10, rural West Bengal), a Nepali survivor, convinced police to lodge an FIR, only to be told to bring a witness and submit documents that her husband was holding. These demands move testimonial injustice into evidentiary impossibility, exploiting the ordinary features of domestic abuse, privacy, isolation and perpetrator control of papers to block redress while presenting refusal as procedural fidelity. In effect, ‘procedure’ operationalises bias by conditioning seriousness on proofs that are systematically unavailable to those most at risk.^74^

These practices form what we might call a credibility economy with three elements. First, typecasting (the ‘misusing complainant’, the ‘false 498A case’, the ‘deviant lesbian’) sets prior bias against belief. Second, triage heuristics (‘weeding out false cases’) convert suspicion into professional virtue, thereby normalising non-registration or diversion as good policing or ‘balanced’ lawyering. Third, anticipatory corroboration establishes evidentiary thresholds that make refusal appear to be compliance with standards rather than abdication of professional responsibility. Taken together, these elements ensure that discounting the survivor’s word is not seen as a refusal; it appears as due diligence.

Analytically, pre-emptive discrediting primes the subsequent dynamics of patriarchal forestalling. Once credibility is discounted, institutional redirection to ‘counselling’, community fora or ‘compromise’ can be rationalised as proportionate and peace-preserving, and procedural dereliction, delay, adjournment and non-enforcement can be narrated as caution rather than neglect. The institutional pathway, therefore, takes shape at the moment of epistemic intake. Anticipatory disbelief does not operate as a provisional stance to be tested; it preselects the institutional route that follows.

### D. Institutional Redirection

Whilst socialisation, compliance and discrediting shape survivors’ internal world, institutional redirection deflects their external recourse. By institutional redirection, I refer to the patterned channelling of survivors away from statutory remedies and into conciliation-oriented forums, such as panchayats, family councils, community bodies and religious authorities, as well as ‘counselling’, where the overriding purpose is to restore cohabitation rather than to secure legal protection. These forums, deeply embedded in patriarchal norms, lack both the mandate and the inclination to deliver gender justice.^75^ Moreover, this redirection is often disguised as culturally appropriate or restorative, though in practice it reabsorbs survivors into the very familial and communal structures from which they sought protection. Ethnographic work on lawyer-free family courts has demonstrated how the everyday adjudication of ‘marital trouble’ privileges compromise and the resumption of domestic life, absorbing rights claims into a moral economy that treats marital continuity as the proper end of intervention.^76^ Commentary on the PWDVA similarly notes that, in practice, ‘counselling’ by state functionaries and NGOs often substitutes for activating the PWDVA’s remedial architecture (time-bound protection, residence and monetary reliefs), a substitution compounded by budgetary and role-clarity deficits.^77^ The state thus performs a symbolic acknowledgement of harm while materially abdicating its duty to intervene.

In the architecture of the framework, this dynamic is thus distinct from relational compliance (which polices conduct through reputational sanction) and from pre-emptive discrediting (which discounts credibility at intake): redirection reframes a legally cognisable wrong as a social problem to be managed rather than as a rights violation to be remedied. The project data illustrates vividly that such a diversion is neither incidental nor exceptional; rather, it is a routine feature of the help-seeking trajectory, repeatedly justified in the idiom of harmony and accommodation, and frequently enacted by officials and intermediaries who style diversion as judiciousness or care.

The project data makes this trajectory explicit. An NGO representative in West Bengal describes the prevailing sequence as ‘first family level, then community level’, with law as a last resort, and sometimes ‘all levels … simultaneously’ (Devyani, NaW03 NGO representative, urban West Bengal). Police practice is central to this redirection. A Tamil Nadu practitioner reported that officers ‘normalise violence and preach the victims to adjust … re-victimising and guilt-tripping’ women (Selvarani, TaN02 NGO representative, urban Tamil Nadu). This is not the role envisioned by the PWDVA and other implementation tools, which emphasise that police should inform survivors of remedies, link them to POs and service providers, and assist with enforcement, not replace these duties with generic ‘counselling’.^78^ Moreover, when ‘assistance’ collapses into reconciliation in this manner, diversion becomes the default script.

This articulation of *law as a last resort* is not simply descriptive; rather, it performs a classificatory move that places reconciliation and adjustment within the domain of virtue and frames rights claiming as premature or excessive. The dataset records survivors ‘weaving in and out’ of family and community remedies, sometimes at the behest of the police, with the form of community mechanisms varying by region and by rural/urban context; in each setting, however, the gravitational pull is towards relational settlement rather than legal remedy. Stakeholders corroborated the central role of state actors in sustaining these channels. Selvarani, an NGO practitioner in Tamil Nadu, described how police practices normalised diversion and reframed persistence as impropriety:

[The p]olice is [the] biggest hurdle (their attitude, beliefs, the power they hold and exercise—in turn re-victimising and guilt tripping the victims—in response they don’t seek for help anymore) [and] threaten with false cases and scare the victims, they normalise violence and preach the victims to adjust the abuse. (TaN02, NGO worker, urban Tamil Nadu)

These accounts align with the project’s broader finding that frontline officers often understood their role as mediation rather than enforcement, encouraging ‘compromise’ and ‘counselling’ instead of registering offences or activating civil protection routes. This is exemplified by Monika (WaPL09 female police officer, urban West Bengal):

They live with the trauma. We try to divert her and pacify her through stories and counsel them. Through counselling, I have been able to reconcile 4–5 couples, and they are living happily now. They came to meet me after that. I feel good that only registering a case is not a solution; resuming domestic life is.

When this occurs, diversion is not an alternative within a menu of options; it *replaces* legal action as the default institutional script.

Redirection is not benign. In rural Tamil Nadu, a panchayat president allegedly proposed a sexual relationship as the condition for ‘support’ (Surya, TcS10, survivor), demonstrating how an abuse of authority can be enabled once claims are moved out of law and into discretionary spaces. Elsewhere, a survivor who approached a women’s police station was directed to the panchayat and then back home ‘with no sanctions’ (Basiakhi, WbS05, semi-urban West Bengal). This passage from a specialised women’s police station to a panchayat and back into the marital home, without protective conditions, is emblematic. The referral is presented as progress; the ‘resolution’ appears orderly, but the violence remains structurally unaddressed. These are not isolated misfires. National monitoring in the *Staying Alive* series documents structural misalignment between the PWDVA’s design and on-the-ground pathways that valorise harmony over remedy. A recent state-level study within a four-state programme similarly records fractured stakeholder networks, protracted procedures and low rates of relief.^79^

Two structural features of redirection deserve emphasis. First, it translates a rights claim into a moral claim about character, casting insistence on protection as evidence of being ‘troublesome’ or ‘disrespectful’ towards community decision makers. This translation supplies reputational costs that are then carried forward into later encounters. Second, it delays justice: by exhausting family and community avenues first, it ensures that legal engagement, when it occurs, is often late and in crisis, thus increasing risk and rendering formal remedies harder to secure. These features explain why redirection, though presented as benign, functions as anticipatory containment within the larger pattern of patriarchal forestalling.

In sum, institutional redirection is best understood as a governance choice rather than a culture-neutral first step. When ‘counselling’ displaces registration, orders and enforcement, the PWDVA’s legal architecture is reabsorbed into the very normative order it sought to regulate, anticipating and containing rights claims before legal standards can be brought to bear.

### E. Procedural Dereliction

Unlike the earlier dynamics of normative socialisation (which sets the moral horizon for complaint), relational compliance (which polices women’s conduct through honour/duty/adjustment), pre-emptive discrediting (which installs a credibility deficit at intake) and institutional redirection (which diverts claims into informal fora), procedural dereliction operates after a case has entered the legal pathway. Its mechanisms are temporal and administrative rather than moral or diversionary: adjournments, missed statutory timelines, partial orders without follow-up, evidentiary dead ends and weak or non-existent enforcement. In this phase, the system preserves recognition but withholds remedy, converting movement on paper into substantive stasis. Put differently, where the earlier stages keep claims from law, procedural dereliction neutralises them within law by making time and procedure do the work of containment. Where diversion does not (or can no longer) contain a claim, procedural dereliction completes the work *inside* legality. Across the three states, survivors who persisted beyond community ‘settlement’ encountered a pathway that appeared active, with files opened, hearings listed and occasional orders drafted; yet protection and relief remained elusive.

The project’s findings evidence this. The PWDVA’s promise of swift civil protection was repeatedly undone by a familiar sequence: legal activation was deferred at the point of entry; cases drifted beyond statutory timelines once they were on the rolls; and even when relief was ordered, enforcement failed to materialise. Each stage preserved the *form* of responsiveness while withholding the *effects* of the remedy. The project data underscores the findings of early and subsequent monitoring, which documented under-resourcing, uneven appointment of POs and delayed or inconsistent orders; these were not episodic faults but recurring features across jurisdictions.^80^ A subsequent state-level study within a four-state programme (Haryana, Maharashtra, Odisha, Tamil Nadu) similarly recorded a ‘dismal’ rate of actual relief, attributing shortfalls to protracted procedures and fractured stakeholder networks.^81^

The project data reveal the same pattern across multiple vantage points. At the point of entry, formal mobilisation was often postponed by inserting extra-legal steps. Police themselves described serial rounds of ‘counselling’ before any FIR or DIR^82^ would be considered—‘Initially we do counselling… two, three, four or even five times before FIR’ (Radhika, WaPL12, female police officer, urban West Bengal)—and defended this displacement as institutional prudence: ‘registering a case is not a solution, resuming domestic life is’ (Monika, WaPL09, female police officer, urban West Bengal). Elsewhere, visible injury was trivialised—‘ignore it since you have to live with the husband’ (Sona, MaS17, survivor, urban Maharashtra)—so that non-registration could be reframed as measured judgment. Entry to the legal pathway, in other words, was itself a site of attrition, screening out those who could not afford delay or the reputational costs of prolonged exposure.

Dereliction also took the form of evidentiary dead ends that made refusal appear like fidelity to procedure. Front-loaded demands for corroboration, witnesses to private abuse and documents controlled by the perpetrator converted the ordinary conditions of domestic violence (privacy, isolation, paper control) into reasons to deny relief. In rural West Bengal, Tapashi (WaPL10), a Nepali survivor, was asked to produce witness statements and paperwork held by her husband; what reads as a neutral proof seeking functioned as exclusion, especially for migrants with precarious status and thin networks.

Once cases did enter the PWDVA channel, time was the main barrier. Stakeholders were unequivocal: a Tamil Nadu lawyer has ‘never known’ the six-month statutory horizon to be met (Deepika, TbL01); a PO observed that cases run ‘much longer than the limit’ (Lubna, TaP01, female PO, urban Tamil Nadu). The Maharashtra sub-sample quantifies the pattern: none of the survivors who initiated proceedings received orders within the time frame; 19 of 25 cases remained pending. For daily-wage earners and women in insecure housing, each adjournment involved lost income, additional travel, childcare disruption and renewed exposure at home. Several survivors responded by recalibrating claims downward as months passed, accepting ‘compromise’ arrangements that secured neither safety nor subsistence. Drift, in this sense, did not simply postpone justice; it reshaped what could be asked for without untenable cost.

Even when orders were issued, implementation faltered. Practitioners were blunt that ‘the implementation of the order is the most frustrating aspect … there are no checks and follow ups’ (Gayatri, TaL02, female lawyer, urban Tamil Nadu). Survivors and lawyers alike identified the same pressure points: service of orders, the absence of compliance reviews, weak breach reporting and cumbersome recovery for monetary reliefs, especially maintenance. The institutional linchpin designed to knit these steps together, POs, was widely judged pivotal but inaccessible: fewer than half of survivors even knew about POs, and those who did frequently struggled to reach an officer or to sustain contact in offices characterised by thin staffing and low visibility. Positive engagements were evident in the data, but as exceptions that highlighted the rule of under-capacity.

Crucially, the experiences of lawyers and the judiciary demonstrate how dereliction is normalised as a professional judgment and a part of courtroom routine. Some lawyers redirected or minimised claims rather than pressing for time-bound relief: ‘What difference does one slap make’ (Amitabh, WaL02, male lawyer, urban West Bengal) typified a stance that recoded harm as trifling and made delay look reasonable. Others invoked ‘misuse’ to rationalise gatekeeping or pathologised claimants (‘emotionally weak’: Pandian, TcL04, male lawyer, rural Tamil Nadu), thereby raising proof thresholds and discouraging robust applications. These lawyerly heuristics have procedural consequences: advice to ‘settle’, to ‘wait, or to ‘seek counselling’ translates directly into adjournments, diluted claims and non-pursuit of breaches.

Inside the courtroom, survivors encountered a conciliatory adjudicatory culture and stratified procedural voice. A practitioner observed that judicial staff and judges could ‘easily silence women from lower sections of society (poor, uneducated women)’ (Sajeev, WaL08, female lawyer, semi-urban West Bengal); disabled women reported inaccessible court infrastructure; and LGBTQI survivors described overt moralisation of their relationships (Rubina, WaS21, survivor, urban West Bengal). These dynamics did not merely produce bad experiences; they generate procedural outcomes, longer cause lists, repeated adjournments to ‘explore settlement’ and tepid responses to breaches that absorb time without increasing safety. Even where orders were issued, the lack of follow-up by judges and staff meant that service, compliance checks and breach recording rarely occurred with the regularity the DWVPA presupposes, leaving monetary and protection orders largely symbolic.

Enforcement gaps are an allied feature. Even where protection or maintenance orders are obtained, execution falters, service, compliance checks, breach reporting and recovery prove cumbersome. Implementation manuals detail PO duties precisely to close these gaps, yet evaluations repeatedly note thin staffing, low visibility and limited training, conditions that undermine both timeliness and enforcement.^83^ Recent judicial directions acknowledge the systemic character of these deficits.^84^ The fact of continued higher court supervision underlines what the empirical record already shows: institutional capacity and procedural design routinely fall short of the PWDVA’s aims.

Lastly, dereliction interacts with the earlier dynamics of the framework. Where pre-emptive discrediting and institutional redirection have already delayed engagement, cases that reach court do so late and precariously. There, adjournments and non-enforcement finish the work: endurance is depleted, claims are trimmed to what seems ‘realistic’ and withdrawal or nominal settlement is retrospectively narrated as choice. The cumulative effect is a system that recognises claims without realising remedy, a temporal arm of containment that absorbs the energy of resistance until it dissipates.

## 6. Conclusion—Law’s Anticipatory Failures and the Myth of Remedy

Building on Dembroff’s theorisation of patriarchy as a distinct, unjust social system, a closer examination of survivors’ journeys through the lens of patriarchal forestalling reveals how patriarchal ideology materially functions as a persistent barrier to justice for women facing domestic violence. Patriarchy is thus not an abstract or outdated concept, but rather a causal structure embedded in social practices and institutional arrangements, designed to reproduce gender hierarchies through iterative loops of normative socialisation. These loops are visible across the trajectories of survivors of domestic violence who sought justice, only to find themselves entangled in systems that systematically devalue their claims, compromise their agency and ultimately leave violence unaddressed. The findings illustrate that justice for many survivors is not a linear progression through the legal system but rather a circuitous path marked by trial and error and disillusionment with both informal and formal institutions. Survivors’ engagements with justice institutions consistently illustrate how the law functions not as a neutral mechanism, but as one shaped by the social system in which it operates.

This phenomenon also reflects the anticipatory logic of the framework, whereby justice systems are structured to contain, neutralise or preclude women’s resistance through layered mechanisms of socialisation, compliance, redirection, discrediting and procedural delay. At the core of the survivors’ experiences is the institutionalisation of patriarchy within both formal and informal mechanisms of justice. Survivors’ accounts illustrate how patriarchy functions not only within institutional responses, but also within the broader social expectations that condition women’s behaviour. Many women delayed seeking help out of fear of social stigma, familial backlash or concern for their children’s futures, all of which reflect internalised patriarchal norms about femininity, motherhood and family duty. Relational compliance ensured that survivors’ lives were monitored and disciplined by family and community members, embedding social norms that rewarded silence and punished resistance, thereby reinforcing subordination through intimate channels of control.

As a result, support spaces became sites of containment, reinforcing adjustment as the rational, culturally acceptable path. These interventions, although often well-intentioned, reinforced the same ideological schema that underlies formal institutions, patriarchy as a structure of gendered endurance and compromise, rather than resistance and redress. This reveals the recursive nature of patriarchal forestalling: survivors may shift domains, from courts to communities, but the normative scripts they encounter remain essentially unchanged.

This was particularly stark in the way panchayats and police officials push women to ‘adjust’ to abuse, trivialise violence and actively discourage divorce. The moral logic of these institutions, which prioritises social harmony, male reputation and family unity over women’s well-being, demonstrates how patriarchy operates through a system of values that delegitimises the grievances of women. For instance, the survivors’ attempts to seek redress are not interpreted as valid demands for justice, but rather as social disruptions to be managed or suppressed. Even well-intentioned intermediaries, such as NGO workers and POs, are rendered ineffective within the larger patriarchal logic that governs the justice system, where peaceful reconciliation is preferred over legal accountability and law enforcement personnel view complaints of violence as standard marital friction.

This illustrates the broader ideological dimension of patriarchal forestalling: the system is designed not only to delay justice, but also to reframe violence as culturally tolerable, resistance as disruptive and reconciliation as the moral endpoint of intervention. Of particular significance is the demonstration of patriarchy as an intersectionally unjust system. The compounded and intersectional marginalisation of LGBTI survivors, disabled women, migrant women and women from lower socio-economic backgrounds points to how patriarchal structures intersect with other axes of oppression. The denial of justice to these women is not incidental but structurally embedded.

The findings also demonstrate the epistemic injustice^85^ within patriarchal systems, in terms of the systemic disbelief of women’s testimony and the denial of credibility to their experiences, disbelief, trivialisation or coercion to reconcile with their abusers. The research findings are replete with examples of women not being believed, being blamed for provoking violence or being told to tolerate abuse, including by female police officers and judges. These institutional behaviours reflect not only how state agents reproduce patriarchal norms through their discretionary decision making and routine practices, but also how procedural dereliction undermines the enforcement of protective laws by exhausting survivors, overburdening them with proof and delaying action in ways that ultimately deter engagement with the system.

The mythologisation of justice as a remote or accidental outcome, achievable only through the rare convergence of trained, empathetic and incorruptible actors, thus emerges as a poignant narrative across the testimonies. Justice is a chimera. Within the architecture of domestic violence law, the Indian example reveals a justice apparatus that is procedurally functional yet substantively inert, where anticipatory logics erode the emancipatory promise of legal remedy.

Patriarchal forestalling thus explains why a formally rights-protective regime can repeatedly deliver *recognition without remedy* and offers a critical vocabulary for comparative engagement with how liberal legal orders, even when formally rights-affirming, can remain deeply enmeshed in gendered hierarchies. Rather than treating implementation gaps as sporadic failure, the framework shows how law anticipates and contains disruption through a sequence of mechanisms that begin outside institutions and culminate within them. Across sites and actors, the outcome is structurally consistent: the legal system acknowledges women’s claims yet withholds their practical effects.

In doing so, it advances a general jurisprudential claim for a legal readership. If the law’s remedial promise can be absorbed into routine administrative practice, then the appropriate unit of analysis is not the rule but the organisational ecosystem that implements it. Forestalling brings into view the moments at which organisational design and professional habit re-encode rights as discretion: when ‘counselling’ replaces registration; when proof thresholds are set at levels that are structurally unattainable; when orders are issued without a compliance infrastructure; and when settlement is treated as the moral endpoint of intervention. These are not outliers; they are predictable consequences of the system’s design. The conceptual contribution of patriarchal forestalling thus travels beyond South Asia; these dynamics are not unique to India. Jurisdictions with ostensibly robust domestic violence laws in the United States^86^ and Europe^87^ reveal strikingly similar patterns of procedural inaction, victim blaming and institutional redirection.

In sum, a recalibration of legal reform efforts is required that is attentive not merely to doctrinal insufficiencies, but to the anticipatory and recursive ways in which patriarchal power is enacted through law. Without dismantling the material and normative structures that sustain patriarchal injustice, within both state and non-state institutions, efforts to reform justice mechanisms will remain partial and insufficient. Survivors’ experiences thus call for a reinvigorated feminist analysis that foregrounds patriarchy as both the context and the cause of their ongoing marginalisation in the pursuit of justice. We must pay attention to patriarchy.

## Footnotes

1 This article draws on data generated by a British Academy-funded project carried out by an academic–civil society research team in Maharashtra, Tamil Nadu and West Bengal (July 2021–April 2022). In India, the lead investigators responsible for conducting the empirical research and co-authoring their respective state reports were: in Maharashtra: Girija Godbole and Preeti Karmarkar; in Tamil Nadu: Swarna Rajagopalan; and in West Bengal: Supurna Banerjee, Nandini Ghosh and Ruchira Goswami. The principal investigators were the author and Professor Phillipa Williams, who co-authored the final all-India report. The author is, however, solely responsible for the analysis and interpretations contained in this article; any errors are mine.

2 UN General Recommendation No 19 *Violence against Women* (CEDAW Committee 1992); see also Rashida Manjoo, ‘Report of the Special Rapporteur on Violence against Women, Its Causes and Consequences’ (UN General Assembly 2013) A/HRC/23/49.

3 Y Ertürk, ‘The Due Diligence Standard: What Does It Entail for Women’s Rights?’ in Carin Benninger-Budel (ed), *Due Diligence and Its Application to Protect Women from Violence* (Brill/Nijhoff 2008).

4 World Bank, *Women, Business and the Law 2021* (2021).

5 Paula Tavares, ‘Protecting Women from Violence—Bridging the Implementation Gap Between Law and Practice’ (World Bank Research and Development Center 2019) Indicators Group Research Note No 4, DOI: 10.13140/RG.2.2.16196.55689.

6 World Bank, *Enforcing Laws on Gender-Based Violence: Implementation Gaps and Barriers to Justice* (2022).

7 ibid.

8 WHO, *Violence Against Women Prevalence Estimates, 2018* (2021) <www.who.int/publications/i/item/9789240022256>.

9 LCWRI, *Staying Alive: Fourth Monitoring & Evaluation Report on the Protection of Women from Domestic Violence Act, 2005* (Lawyers Collective 2013).

10 Anamika Das and CM Lakshmana, ‘The Implementation of Domestic Violence Act in India: A State-Level Analysis’ (Institute for Social and Economic Change 2020) Working Paper No 499.

11 On legal institutionalism and the epistemic framing of legal authority, see David Dyzenhaus, *The Constitution of Law: Legality in a Time of Emergency* (CUP 2006) esp ch 2; Elizabeth Fisher, ‘Legal Reason, Administrative Capacity, and Deep Structure in the Regulatory State’ (2013) 36 UNSWLJ 446.

12 Across the state reports, the study’s intersectional sampling approach is evidenced primarily through sample-profile tables, although the level of detail reported varies by state. In Maharashtra, the sample is numerically disaggregated by religion (Hindu, 40; Buddhist, 12; Muslim, 5; Other, 3) and caste (General, 25; SC, 16; OBC, 7; NT, 6; ST, 4; EBC, 1; DT, 1), and class is reported using employment status as a proxy (Employed/salaried, 21; Housewife only, 14; Informal work, 8; Casual labourer, 7; Other, 6; Entrepreneur/own account, 4). In West Bengal, the report similarly provides numeric disaggregation by religion (Hindu, 44; Muslim, 8; Christian, 5; Buddhist, 2; Other, 1) and caste (General, 36; SC, 11; ST, 2; OBC, 11), with occupation used as a proxy for class (Casual labourer, 13; Salaried, 9; Business owner/own account, 8; Housewife only, 12; Informal work, 9; Other, 9). West Bengal additionally records disability status in the sample (five disabled survivors) and notes inclusion beyond women (58 women and two non-binary participants). Whilst the West Bengal narrative indicates inclusion of lesbian and bisexual participants, a numeric distribution of the survivor sample by sexual orientation and disability is not enumerated in the Maharashtra tables.

13 WHO, ‘Putting Women First: Ethical and Safety Recommendations for Research on Domestic Violence against Women’ (2001) <www.who.int/publications/i/item/WHO-FCH-GWH-01.1> accessed 17 March 2026. For a detailed overview of the application of research ethics to the study and its participants, see the project report here: https://www.survivingviolence.org/resources/ accessed 17 March 2026.

14 International Institute for Population Sciences, *National Health Family Survey (NFHS-5), 2019–21* (IIPS 2021) <https://dhsprogram.com/pubs/pdf/FR375/FR375.pdf> accessed 17 March 2026.

15 National Crime Records Bureau (NCRB), *Crime in India 2022 Statistics* (Ministry of Home Affairs, Government of India 2023).

16 <www.ncw.gov.in/publications/reports-published-by-the-commission/> accessed 17 March 2026.

17 A Basu, ‘Building Strong Alliances: The Politics of the Protection of Domestic Violence Act in India (2006)’ in S Nazneen, S Hickey and E Sifaki (eds), *Negotiating Gender Equity in the Global South: The Politics of Domestic Violence Policy* (Routledge 2019).

18 There are now new criminal codes in India, introduced in 2023. This provision is now replicated in sections 84–6 of the new Bharatiya Nyaya Sanhita.

19 Srimati Basu, *The Trouble with Marriage: Feminists Confront Law and Violence in India* (University of California Press 2015); P Roychowdhury, *Capable Women, Incapable States: Negotiating Violence and Rights in India* (OUP 2021).

20 Protection of Women from Domestic Violence Act (2005) <https://www.indiacode.nic.in/bitstream/123456789/15436/1/protection_of_women_from_domestic_violence_act%2C_2005.pdf> accessed 17 March 2026.

21 s 498A of the IPC is a criminal law that protects married women from their spouses and the relatives of the spouse from inflicting cruelty on women.

22 See n 17.

23 See eg B Ghosh and T Choudhuri, ‘Legal Protection against Domestic Violence in India: Scope and Limitations’ (2011) 26 Journal of Family Violence 319; Lawyers Collective (n 9); Audrey D’Mello and Flavia Agnes, ‘Protection of Women from Domestic Violence’ (2015) 50(44) Economic and Political Weekly 76; Roychowdhury (n 19); S Nigam, *Women and Domestic Violence Law in India: A Quest for Justice* (Routledge India 2019).

24 Oxfam, ‘Towards Violence Free Lives for Women: Tracking of Union Budgets (2018–21) for Violence Services’ (2021) <www. oxfamindia.org/knowledgehub/workingpaper/ towards-violence-free-lives-women-tracking-union-budgets-2018-21-violence-services> accessed 17 March 2026.

25 Press Information Bureau, ‘Protection Officers under PWDVA (2005)’ (Ministry of Women and Child Development, Government of India 2013) <https://www.pib.gov.in/newsite/PrintRelease.aspx?relid=92846&reg=3&lang=2> accessed 17 March 2026

26 S Banerjee and others, *The Violent Domestic: Law, Its Practice and Strategies of Survival* (Zubaan Academic 2022).

27 NCRB, *Crime in India 2023, Statistics Volume 1* (Ministry of Home Affairs, Government of India 2024) <www.scribd.com/document/942536771/1CrimeinIndia2023PartI1> accessed 17 March 2026.

28 ‘Supreme Court Criticizes Delays in Domestic Violence Cases, Calls for Swift Implementation of PWDVA’ (*The Legal Affair*, 5 November 2024).

29 Noting the dismal situation, the court issued several directives to address the systemic challenges facing the implementation of the PWDVA, including the undertaking of an empirical study in collaboration with the state to assess the inadequacy of POs and other resources.

30 Roychowdhury (n 19). She argues that the state has effectively outsourced its responsibility to civil society organisations, which have long focused on women’s rights and gender violence issues and perceive their responsibility as being to inform the law, as well as to cultivate and empower ‘capable’ women to navigate legal justice mechanisms.

31 This question asked ‘if they agree that a husband is justified in hitting or beating his wife under each of the following seven circumstances: she goes out without telling him, she neglects the house or the children, she argues with him, she refuses to have sex with him, she doesn’t cook food properly, he suspects her of being unfaithful, and she shows disrespect for her in-laws’ (NFHS-5 2019/21).

32 See n 14.

33 S Kanougiya and others, ‘Prevalence, Pattern, and Predictors of Formal Help-Seeking for Intimate Partner Violence against Women: Findings from India’s Cross-Sectional National Family Health Surveys-3 (2005–2006) and 4 (2015–2016)’ (2022) 22 BMC Public Health 2386.

34 See n 14. NFHS-5 data capture self-reported experiences of domestic violence and help-seeking behaviour, while NCRB statistics record only police-registered cases and court outcomes. NCRB reporting on the PWDVA is limited to criminal prosecutions for breach of protection orders and does not reflect the volume or effectiveness of civil relief granted under the PWDVA. As a result, NCRB figures should be read as indicators of criminal justice system engagement and enforcement, rather than as measures of prevalence.

35 See n 27.

36 See eg Sally Engle Merry, *Human Rights and Gender Violence: Translating International Law into Local Justice* (University of Chicago Press 2006); S Nigam, *Domestic Violence Law in India: Myth and Misogyny* (Routledge India 2021).

37 Sally Haslanger, ‘Social Systems and Intersectional Oppression’ in Ruth Chang and Amia Srinivasan (eds), *Conversations in Philosophy, Law, and Politics* (OUP 2024) 399.

38 Haslanger argues that unjust social systems are self-perpetuating because they constrain individual and collective agency through institutional roles, normative expectations and ideological reinforcement. These constraints compel individuals to act in ways that reproduce the very structures that limit them, often without intention or awareness. See Sally Haslanger, *Resisting Reality: Social Construction and Social Critique* (OUP 2012) esp 226–35, 331–41.

39 As noted by Robin Dembroff, ‘Intersection Is Not Identity, or How to Distinguish Overlapping Systems of Injustice’ in Chang and Srinivasan (n 37) 383.

40 ibid.

41 Sylvia Walby, ‘Theorising Patriarchy’ (1989) 23(2) Sociology 213; Bell Hooks, *Feminism Is for Everybody* (South End Press 2000); Catharine MacKinnon, *Toward a Feminist Theory of the State* (Harvard UP 1989).

42 Robin Dembroff, ‘Real Talk on the Metaphysics of Gender’ (2018) 46(2) Philosophical Topics 21.

43 In response to Haslanger’s argument that patriarchy does not exist as a system in itself. See Haslanger, ‘Why I Don’t Believe in Patriarchy: Comments on Kate Manne’s Down Girl’ (2020) 101(1) Philosophy and Phenomenological Research 220.

44 Nivedita Menon, *Recovering Subversion: Feminist Politics Beyond the Law* (University of Illinois Press 2004); Flavia Agnes, ‘Law, Ideology and Female Sexuality: Gender Neutrality in Rape Law’ (2002) 37(9) Economic and Political Weekly 844; R Kapur, *Erotic Justice: Law and the New Politics of Postcolonialism* (Routledge-Cavendish 2005).

45 Martha Fineman, *The Autonomy Myth: A Theory of Dependency* (The New Press 2004); see also Amia Srinivasan, ‘The Aptness of Anger’ (2018) 31(3) Journal of Political Philosophy 355.

46 D Kandiyoti, ‘Bargaining with Patriarchy’ (1988) 2(3) Gender & Society 274; Patricia Uberoi, ‘The Family in India: Beyond the Nuclear versus Joint Debate’ in Veena Das (ed), *The Oxford Companion to Sociology and Social Anthropology* (OUP 2003) 1073.

47 Robert W Connell, ‘Theorising Gender’ (1985) 19 Sociology 260.

48 Kandiyoti (n 46); Uberoi (n 46) 1073.

49 See Banerjee and others (n 26); Veena Das, *Critical Events: An Anthropological Perspective on Contemporary India* (OUP 1996).

50 Flavia Agnes, *Law and Gender Inequality: The Politics of Women’s Rights in India* (OUP 2001); Rajesh Kumar Singh, ‘Barriers to Accessing Legal Aid for Domestic Violence Victims in Rural India: A Study of Uttar Pradesh’ (2024) 3(9) Studies in Social Science & Humanities 31.

51 S Krishnan, ‘Gender, Caste, and Economic Inequalities and Marital Violence in Rural South India’ (2005) 26(1) Health Care for Women International 87; S Rukmini, *Whole Numbers and Half Truths: What Data Can and Cannot Tell Us About Modern India* (Context/Westland 2021).

52 See Shireen J Jejeebhoy, ‘Wife-Beating in Rural India: A Husband’s Right? Evidence from Survey Data’ (1998) 33(15) Economic and Political Weekly 855; Leela Visaria, ‘Violence against Women in India: Evidence from Rural Gujarat’ in *Domestic Violence in India: A Summary Report of Three Studies* (International Center for Research on Women 1999) 14–25.

53 BS Nayak and G Sinha, ‘Patriarchal Family, Gendered Society and Capitalist State in India’ in BS Nayak and N Tabassum (eds), *Impact of Patriarchy and Gender Stereotypes on Working Women. Diversity and Inclusion Research* (Springer 2024).

54 Ann Swidler, ‘Culture in Action: Symbols and Strategies’ (1986) 51 American Sociological Review 273.

55 U Chakravarti and M Krishnaraj, *Gendering Caste* (SAGE Publications 2018).

56 Merry (n 36).

57 ibid; Basu, *The Trouble with Marriage* (n 19).

58 Uma Chakravarti, *Gendering Caste: Through a Feminist Lens* (Stree 2003); Prem Chowdhry, *Contentious Marriages, Eloping Couples: Gender, Caste, and Patriarchy in Northern India* (OUP 2007).

59 A traditional village council in India. It is meant is a form of local self-government in India where community members come together to make decisions.

60 Feminist socio-legal work on family courts in India similarly documents how the institutional language of ‘compromise’ and ‘reconciliation’ draws on this moral economy, recasting protection as the resumption of domestic life. Basu, *The Trouble with Marriage* (n 19) chs 2–4 (on mediation, reconciliation and compromise in family-court practice).

61 Veena Das, *Life and Words: Violence and the Descent into the Ordinary* (University of California Press 2007).

62 Kandiyoti (n 46).

63 Setyorini Setyorini and others, ‘Defying the Odds: Can Women Truly Thrive in a Patriarchal World?’ (2024) 46(4) Journal of Public Health.

64 A Irudayam, JP Mangubhai and J Lee, *Dalit Women Speak Out: Violence against Dalit Women in India* (Zubaan 2014); Gopal Guru, *Humiliation: Claims and Contexts* (OUP 2009).

65 S Arora and M Majumder, ‘Where Is My Home? Gendered Precarity and the Experience of COVID-19 among Women Migrant Workers from Delhi and National Capital Region, India’ (2021) 28(Suppl 2) Gender, Work & Organization 307; S Kumari, E Kour and A Sharma, ‘Women at Margin: Exploring the Vulnerability of Women in Unorganised Sector of Jammu City’ (2024) 62 The Deccan Geographer 47.

66 Miranda Fricker, *Epistemic Injustice: Power and the Ethics of Knowing* (OUP 2007); José Medina, *The Epistemology of Resistance: Gender and Racial Oppression, Epistemic Injustice, and Resistant Imaginations* (OUP 2013).

67 Suruchi Thapar Björkert, ‘Women as Arm-Bearers: Gendered Caste-Violence and the Indian State’ (2006) 29(5) Women’s Studies International Forum 474; J Khubchandani and others, ‘Caste Matters: Perceived Discrimination among Women in Rural India’ (2018) 21(2) Archive of Women’s Mental Health 163.

68 See *Arnesh Kumar v State of Bihar* (2014) 8 SCC 273; *Rajesh Sharma v State of Uttar Pradesh* (2017) 8 SCC 821 (as modified in *Social Action Forum for Manav Adhikar v Union of India* (2018) 10 SCC 443).

69 Most of these cases have been about extended family members being implicated. See *Dara Lakshmi Narayana v. State of Telangana* (2025) 3 SCC 735; *Mange Ram v State of Madhya Pradesh* 2025 INSC 962; *Shobhit Kumar Mittal v State of Uttar Pradesh* 2025 INSC 1152.

70 2025 INSC 536, para 11.

71 Empirical socio-legal work on family court practice in India similarly documents how routine suspicion of women’s motives, particularly around maintenance and custody, structures the everyday triage of cases. See Basu, *The Trouble with Marriage* (n 19) chs 2–4.

72 Pratiksha Baxi, *Public Secrets of Law: Rape Trials in India* (OUP 2014) esp chs 3–4 (on courtroom credibility, injury and humiliation). See also Rupal Oza, *Semiotics of Rape: Sexual Subjectivity and Violation in Rural India* (Duke UP 2022).

73
*National Legal Services Authority v Union of India* (2014) 5 SCC 438.

74 Fricker (n 66).

75 Nirmala Buch, ‘Women’s Experience in New Panchayats: The Emerging Leadership of Rural Women’ (Centre for Women's Development Studies 2009) Occasional Paper No 35; Prem Chowdhry, ‘Crisis of Masculinity in Haryana: The Unmarried, the Unemployed and the Aged’ (2005) 40(49) Economic and Political Weekly 5189.

76 Basu, *The Trouble with Marriage* (n 19) chs 1–3.

77 D’Mello and Agnes (n 23).

78 LCWRI, *Ending Domestic Violence Through Non-Violence: A Manual for PWDVA Protection Officers* (Lawyers Collective 2010) <https://lawyerscollective.org/wp-content/uploads/2023/04/Protection-officers-manual.pdf> accessed 17 March 2026; see also RCI-VAW/TISS–ILS stakeholder manuals <https://projecttech4dev.org/resource-centre-for-interventions-on-violence-against-women-tiss/> accessed 17 March 2026.

79 Monica Sakhrani and others, ‘Long Road to Justice: Implementation of the Protection of Women from Domestic Violence Act (2005) in Haryana’ in Indira Jaising and Pinki Mathur Anurag (eds), *Conflict in the Shared Household: Domestic Violence and the Law in India* (OUP 2019).

80 LCWRI, *Staying Alive*, n 9.

81 Sakhrani and others (n 79).

82 Domestic Incident Report.

83 LCWRI, *Ending Domestic Violence* (n 78); see also RCI-VAW/TISS–ILS stakeholder manuals (n 78).

84 On 20 May 2025, the Supreme Court directed all states and union territories to identify and designate POs to ensure the proper implementation of the PWDVA in *We the Women of India v Union of India* Note 23.

85 Fricker (n 66).

86 See eg Leigh Goodmark, *Decriminalizing Domestic Violence: A Balanced Policy Approach to Intimate Partner Violence* (University of California Press 2018).

87 See eg Shazia Choudhry, ‘When Women’s Rights Are “Not” Human Rights—the Non-Performativity of the Human Rights of Victims of Domestic Abuse within English Family Law’ (2019) 82 MLR 1072 <www.jstor.org/stable/45284618> accessed 12 September 2025.

